# 46,XX males with congenital adrenal hyperplasia: a clinical and biochemical description

**DOI:** 10.3389/fendo.2024.1410122

**Published:** 2024-08-08

**Authors:** Bas P. H. Adriaansen, Agustini Utari, Dineke Westra, Achmad Zulfa Juniarto, Mahayu Dewi Ariani, Annastasia Ediati, Mariska A. M. Schröder, Paul N. Span, Fred C. G. J. Sweep, Stenvert L. S. Drop, Sultana M. H. Faradz, Antonius E. van Herwaarden, Hedi L. Claahsen – van der Grinten

**Affiliations:** ^1^ Department of Pediatrics, Division of Pediatric Endocrinology, Amalia Children’s Hospital, Radboud University Medical Center, Nijmegen, Netherlands; ^2^ Department of Laboratory Medicine, Radboud University Medical Center, Nijmegen, Netherlands; ^3^ Center for Biomedical Research, Faculty of Medicine, Diponegoro University, Semarang, Indonesia; ^4^ Department of Pediatrics, Division of Pediatric Endocrinology, Faculty of Medicine, Diponegoro University, Semarang, Indonesia; ^5^ Department of Human Genetics, Radboud University Medical Center, Nijmegen, Netherlands; ^6^ Faculty of Psychology, Diponegoro University, Semarang, Indonesia; ^7^ Department of Radiation Oncology, Radboud University Medical Center, Nijmegen, Netherlands; ^8^ Department of Pediatrics, Division of Endocrinology, Sophia Children’s Hospital, Erasmus Medical Center, Rotterdam, Netherlands

**Keywords:** congenital adrenal hyperplasia, 21-hydroxylase deficiencyy, 11-hydroxylase deficiency, glucocorticoid, androgen, virilization, 46,XX males, treatment

## Abstract

**Introduction:**

Congenital adrenal hyperplasia (CAH) due to 21-hydroxylase deficiency (21OHD) or 11-hydroxylase deficiency (11OHD) is characterized by underproduction of cortisol and overproduction of adrenal androgens. These androgens lead to a variable degree of virilization of the female external genitalia in 46,XX individuals. Especially in developing countries, diagnosis is often delayed and 46,XX patients might be assigned as males. This study aims to describe the clinical and biochemical characteristics of a unique cohort of untreated male-reared 46,XX classic CAH patients from Indonesia and discusses treatment challenges.

**Methods:**

Nine untreated classic CAH patients with 46,XX genotype and 21OHD (n=6) or 11OHD (n=3), aged 3-46 years old, were included. Biometrical parameters, clinical characteristics, and biochemical measurements including glucocorticoids, renin, androgens, and the pituitary-gonadal axis were evaluated.

**Results:**

All patients had low early morning serum cortisol concentrations (median 89 nmol/L) without significant increase after ACTH stimulation. Three patients with salt wasting 21OHD reported one or more periods with seizures and/or vomiting in their past until the age of 6, but not thereafter. The remaining patients reported no severe illness or hospitalization episodes, despite their decreased capacity to produce cortisol. In the 21OHD patients, plasma renin levels were elevated compared to the reference range, and in 11OHD patients renin levels were in the low-normal range. All adult patients had serum testosterone concentrations within the normal male reference range. In 21OHD patients, serum 11-oxygenated androgens comprised 41-60% of the total serum androgen concentrations. Glucocorticoid treatment was offered to all patients, but they refused after counseling as this would reduce their endogenous androgen production and they did not report complaints of their low cortisol levels.

**Discussion:**

We describe a unique cohort of untreated classic 46,XX male CAH patients without overt clinical signs of cortisol deficiency despite their cortisol underproduction and incapacity to increase cortisol levels after ACTH stimulation. The described adolescent and adult patients produce androgen levels within or above the normal male reference range. Glucocorticoid treatment will lower these adrenal androgen concentrations. Therefore, in 46,XX CAH patients reared as males an individual treatment approach with careful counseling and clear instructions is needed.

## 1 Introduction

**Figure 1:**
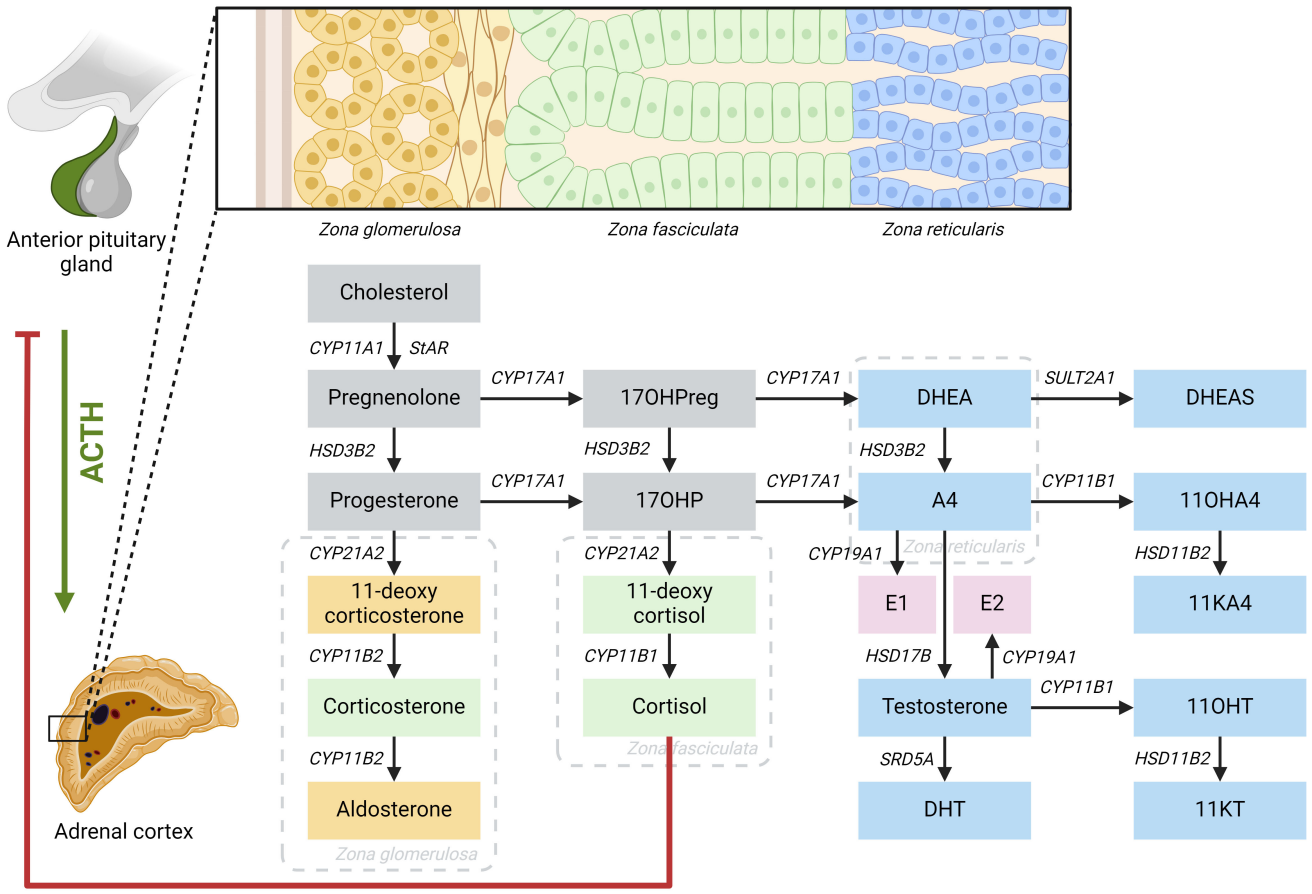
Schematic overview of the steroidogenesis. Yellow boxes: mineralocorticoids; green boxes: glucocorticoids; blue boxes: androgens; pink boxes: estrogens. 11OHA4, 11-hydroxyandrostenedione; 11OHT, 11-hydroxytestosterone; 11KA4, 11-ketoandrostenedione; 11KT, 11-ketotestosterone; 17OHP, 17-hydroxyprogesterone; 17OHPreg, 17-hydroxypregnenolone; A4, androstenedione; ACTH: adrenocorticotropic hormone; *CYP11A1*, cholesterol side-chain cleavage; *CYP11B1*, 11β-hydroxylase; *CYP11B2*, aldosterone synthase; *CYP17A1*, 17α-hydroxylase/17,20-lyase; *CYP19A1*, aromatase; *CYP21A2*, 21α-hydroxylase; DHEA, dehydroepiandrosterone; DHEAS, dehydroepiandrosterone sulphate; DHT, dihydrotestosterone; E1, estrone; E2, estradiol; *HSD3B2*, 3β-hydroxysteroid dehydrogenase type 2; *HSD11B2*, 11β-hydroxysteroid dehydrogenase type 2; *HSD17B*, 17β-hydroxysteroid dehydrogenase; *SRD5A*, 5α- reductase; StAR, steroidogenic acute regulatory protein; *SULT2A1*, sulfotransferase family 2A member 1.

**Figure 2:**
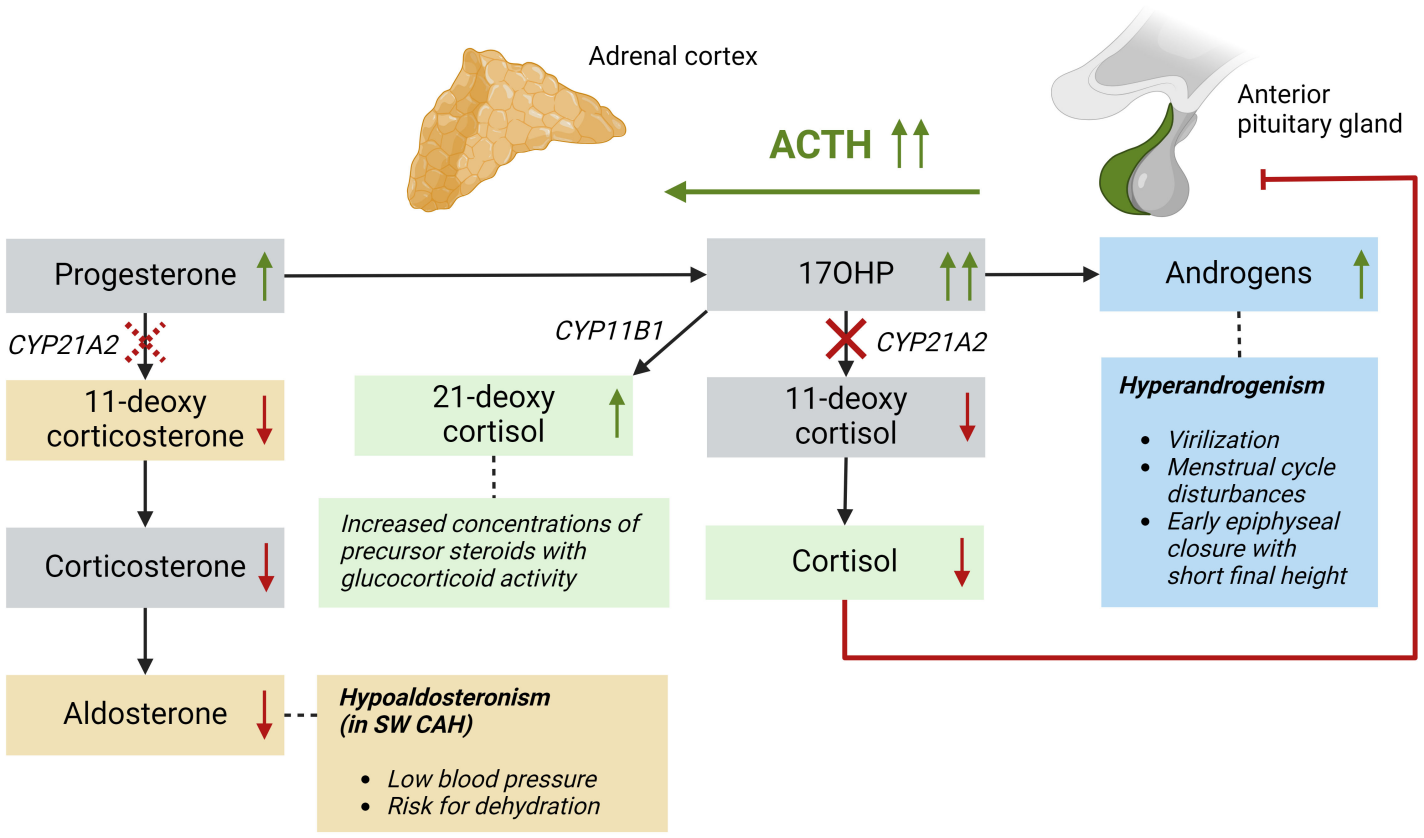
Patients with a 21α-hydroxylase deficiency have impaired conversion of 17OHP into 11-deoxycortisol, also leading to low cortisol levels and subsequently high ACTH levels. High 17OHP levels are converted into 21-deoxycortisol, which has glucocorticoid activity, and into adrenal androgens causing hyperandrogenism. Patients with salt-wasting 21OHD also have impaired conversion of progesterone into 11-deoxycorticosterone leading to low aldosterone levels. This is represented by a dashed line because simple virilizing patients do not have a clinically significant impaired conversion in this mineralocorticoid pathway. ACTH, adrenocorticotropic hormone; *CYP11B1*, 11β-hydroxylase; *CYP21A2*, 21α-hydroxylase; SW, salt-wasting.

**Figure 3:**
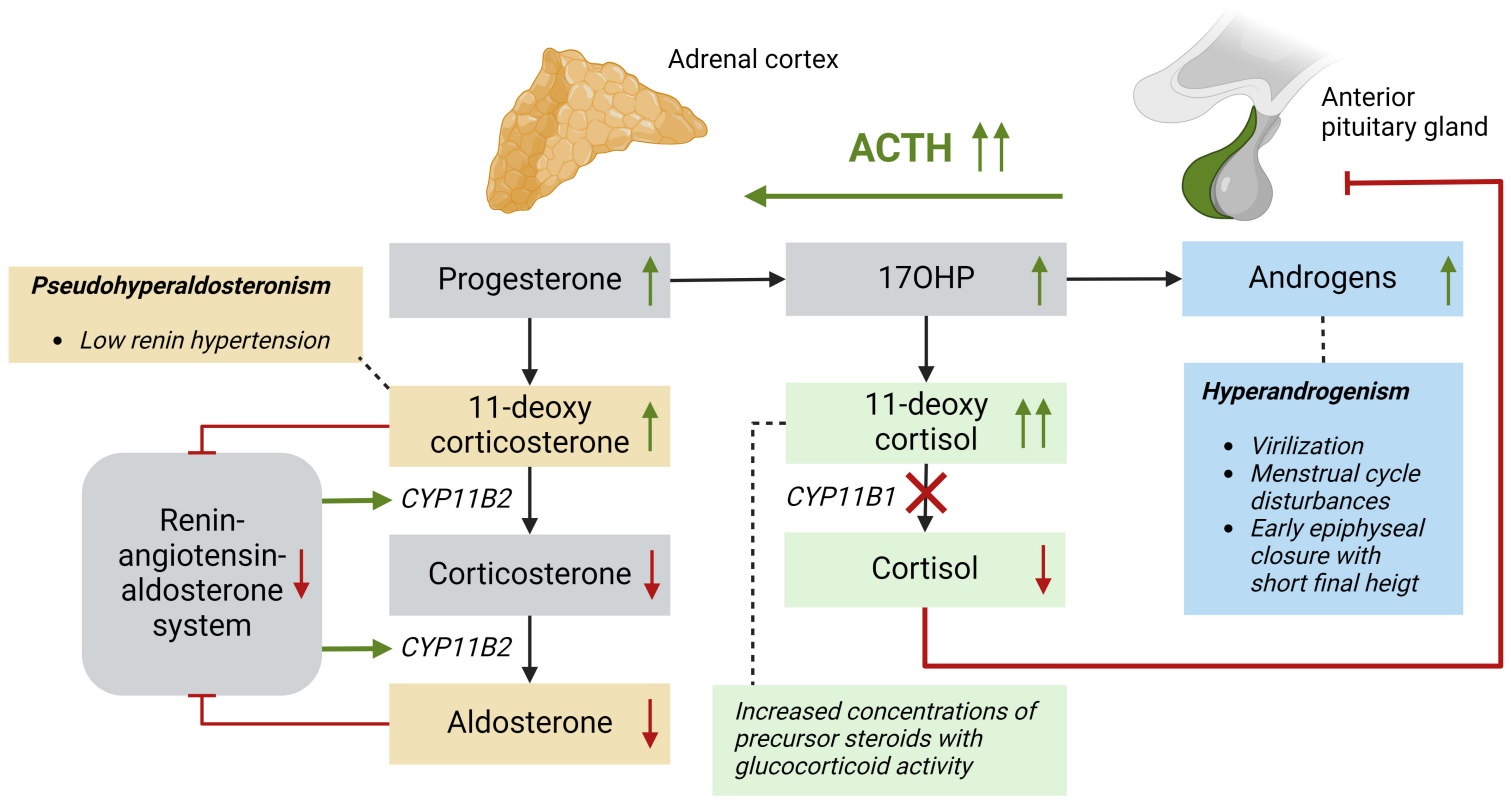
Patients with a 11β-hydroxylase deficiency based on *CYP11B1* mutations have impaired conversion of 11-deoxycortisol into cortisol resulting in low cortisol levels and subsequently high ACTH levels. Precursor steroids, such as 17OHP prior to the enzymatic block increase and are converted into adrenal androgens leading to hyperandrogenism. The mineralocorticoid pathway in these patients is ACTH-dependent, because 11-deoxycorticosterone is produced in high levels and this precursor steroid has high mineralocorticoid activity. These elevated levels lead to negative feedback on the renin-angiotensin-aldosterone system, leading to low renin levels and subsequently less *CYP11B2* activity, which further increases the 11-deoxycorticosterone concentration, aggravating the signs of pseudo hyperaldosteronism. ACTH, adrenocorticotropic hormone; *CYP11B1*, 11β-hydroxylase; *CYP11B2*, aldosterone synthase.

Adrenal steroid hormones like cortisol, aldosterone, and androgens are produced in the adrenal cortex by several enzymatic steps (**Figure 1**). Defects of enzymes involved in adrenal steroidogenesis lead to congenital adrenal hyperplasia (CAH), a group of autosomal recessive disorders. The most common enzymatic defect (>90%) is caused by pathogenic variants in the *CYP21A2* gene, leading to 21α-hydroxylase deficiency (21OHD) (1). This enzyme is involved in the production of both cortisol and aldosterone in the adrenal cortex, and the residual enzymatic activity determines the severity of the disease (2). In the most severe classic form of 21OHD with <1% residual enzyme activity, insufficient concentrations of aldosterone is produced, resulting in a salt-wasting (SW) phenotype (**Figure 2**). In patients with 1 – 2% residual 21α-hydroxylase activity, aldosterone production is secured, and these patients are grouped as the simple virilizing (SV) form (1). The second most common defect is 11β-hydroxylase deficiency (11OHD) caused by pathogenic variants in the *CYP11B1* gene. This gene is solely involved in adrenal cortisol production, and not in the aldosterone production. Patients with 11OHD produce high levels of precursor steroids with mineralocorticoid activity that could lead to hypertension at a young age (3) (**Figure 3**). In both classic 11OHD and classic 21OHD cortisol production is diminished. Therefore, CAH is described as the most important congenital cause of adrenal insufficiency.

Due to a decreased negative feedback by cortisol, the pituitary gland chronically overproduces adrenocorticotropic hormone (ACTH), leading to continuous stimulation of the adrenal cortex (hyperplasia) with elevated levels of precursor steroids prior to the enzymatic block (**Figures 2**, **3**). These precursor steroids are partly shunted into the unaffected adrenal androgen pathway, resulting in elevated concentrations of adrenal androgens like dehydroepiandrosterone (DHEA) and androstenedione (A4). These androgens have relatively low androgenic activity and can be converted into more potent androgens, such as testosterone (T) and dihydrotestosterone (DHT), mainly in peripheral tissues. Besides this classical androgen pathway, an alternative pathway called ‘the backdoor pathway’ can lead to the formation of DHT without T as an intermediate (4). The adrenal androgens A4 and T can be hydroxylated by adrenal-specific 11-hydroxylase into 11-hydroxyandrostenedione (11OHA4) and 11-hydroxytestosterone (11OHT), which can be further metabolized into 11-ketoandrostenedione (11KA4) and 11-ketotestosterone (11KT), respectively (5). This 11KT has similar agonistic activity on the androgen receptor as T (5–8). In 46,XX 21OHD and 11OHD patients, elevated concentrations of adrenal androgens cause a variable degree of virilization of the female external genitalia, ranging from mild clitoromegaly to complete fusion of the labioscrotal walls, possibly leading to male sex assignment after birth (9, 10).

Thus, the hallmark of CAH is not only a decreased production of cortisol, but also an accumulation of precursor steroids and adrenal androgens due to chronic overstimulation of the adrenal cortex by ACTH. Therefore, the current guideline recommends to the substitute for decreased cortisol levels and suppress the elevated ACTH levels to subsequently lower adrenal androgen production (11). To adequately suppress adrenal androgen production, supraphysiological dosages of glucocorticoids are necessary (12). In Western countries, classic 46,XX CAH patients are most often diagnosed and treated from the neonatal period and generally assigned as female immediately after birth. In countries without neonatal screening, sufficient diagnostic infrastructure, and adequate medication, CAH patients are often detected after the first years of life and treatment is delayed. Undiagnosed 46,XX patients with CAH may be assigned as males after birth and have a male sex of rearing (3, 13–15).

Our study describes clinical and biochemical characteristics of nine untreated 46,XX classic CAH patients reared as males. We focus on glucocorticoids, renin, the pituitary-gonadal axis, and both classic and 11-oxygenated androgens, and discuss the challenges in hormonal treatment of these 46,XX males.

## 2 Materials and methods

### 2.1 Patients

We selected patients from a local database at the Center for Biomedical Research (CEBIOR), Diponegoro University, Semarang, Indonesia, containing 60 CAH patients. Of these patients, 22 (all 46,XX genotype) were not treated with glucocorticoids (16) of whom nine were reared as males (aged 3 to 46 years old). The study protocol was approved by the ethical committee of the faculty of medicine, Diponegoro University, Semarang, Indonesia (number 713/EC/FK-RSDK/2016). Written and oral informed consent was obtained from all patients and/or parents. The diagnosis of CAH was confirmed by clinical, hormonal, and genetic analyses. Clinical data were obtained by an experienced pediatric endocrinologist (AU) and included medical history, physical examination, biometry, pubertal status, signs of hyperandrogenism (at gingiva, palm of the hand, and genitalia), and appearance of external genitalia. Bone aged was assessed in pediatric patients according to the atlas of Greulich and Pyle. Patients with 21OHD were phenotypically classified as salt wasting as they were at least once hospitalized for a salt wasting crisis.

### 2.2 Genetic analysis

For patient 1 and 2, genetic analysis was performed as previously described (17). For the remaining patients, we performed molecular analysis of the *CYP21A2* and *CYP11B1* genes in the genome diagnostic laboratory of the Radboud University Medical Center, the Netherlands. Genomic DNA was extracted from peripheral blood leukocytes of the patients according to standard procedures in Indonesia (18) and shipped to the Netherlands. *CYP21A2* gene molecular analysis was performed according to best practice guidelines (19) with PCR-based Sanger sequencing analysis of all ten exons and exon/intron boundaries of the *CYP21A2* gene (NM_000500.7) and MLPA analysis (P050 CAH probe mix, MRC Holland, Amsterdam, the Netherlands) for copy number detection. For *CYP11B1* gene molecular analysis, nine coding exons and flanking parts of the intronic sequences of the *CYP11B1* gene (NM_000497.3) were analyzed with PCR-based Sanger sequencing. Patients were classified as SV and SW based on genotype and clinical evaluation.

### 2.3 Biochemical analyses

An ACTH stimulation test was performed in all patients in the morning before 9.00 a.m. after overnight fasting. Blood was taken before and 60 minutes after a 250 µg synacthen^®^ iv injection (Sigma Tau BV, Utrecht, the Netherlands) following a standard protocol (20). Blood samples were immediately centrifuged (10 minutes, 1500g, 16°C) and serum and EDTA plasma were stored at -80°C until shipment. The specimens were transported on dry ice to the Radboud University Medical Center, the Netherlands. All measurements were performed with clinically validated measuring methods in an ISO15189 accredited laboratory. Serum progesterone, 17OHP, 11-deoxycorticosterone, corticosterone, 11-deoxycortisol, 21-deoxycortsiol, cortisol, estradiol, and estrone were measured by liquid-chromatography tandem mass spectrometry (LC-MS/MS). DHEA, A4, T, DHT, 11OHA4, 11KA4, 11OHT, and 11KT were measured in serum by LC-MS/MS as previously described (5). Free (i.e., unbound) T was measured in serum by LC-MS/MS after *in vitro* dialysis and divided by simultaneously measured total T concentrations to calculate the free T fractions. Renin concentrations were measured in EDTA plasma using immuno radio metric assay (CISbio, gen III Codolet, France). Luteinizing hormone (LH), follicle-stimulating hormone (FSH), and sex hormone binding globulin (SHBG) were measured in serum using electro chemiluminescence immunoassay on an E801 random access analyzer (Roche).

## 3 Results

### 3.1 Clinical description of the patients

Nine 46,XX CAH patients with a male sex of rearing from six non-consanguineous families were included (**Table 1**). Six patients were diagnosed with classic 21OHD and three with 11OHD. The age of the patients at investigation varied from 3 to 46 years old, and none of them ever received daily glucocorticoid treatment. All patients had a below-average height (median z-score -2.97; range -4.19 to -0.34) in comparison to the WHO growth charts. In the two adult patients (patient 2 and 3) the final height was 142.8 cm and 141.1 cm, which is significantly lower than their mid parental height. Bone age (BA) was assessed in five out of seven pediatric patients. All five had increased BA compared to their calendar age; BA advancement ranged from +1.0 to +7.9 years. Patient 4 with a calendar age of 10.1 years already had an adult BA, indicating that his height will not increase further with a final height of 135.9 cm, which is far below his mid parental height (155.0 cm).

**Table 1:** Clinical characteristics of nine untreated 46,XX congenital adrenal hyperplasia patients with a male sex of rearing.

Characteristic	*CYP21A2* deficiency						*CYP11B1* deficiency		
Patient number	1*	2*	3	4	5	6	7**	8**	9**
**Age (years)**	15.6	32.6	46.5	10.1	14.3	15.3	3.5	5.7	13.0
**Phenotype prediction**	SV	SV	SV	SW	SW	SW	Classic	Classic	Classic
**Weight (kg)**	49.1	58.6	49.0	40.3	42.5	50.4	12.2	18.9	30.0
**Height (cm)**	142.5	142.8	141.1	135.9	131.4	146.0	90.3	108.4	135.0
**Z-score (height for age)**	-2.97	-3.11	-3.37	-0.34	-4.19	-2.34	-2.36	-1.19	-3.18
**Body mass index (kg/m^2^)**	24.1	28.7	24.6	21.8	24.6	23.6	14.96	16.5	16.1
**Mid parental height**	149.5	149.5	157.5	155.0	147.8	149.5	146.1	146.1	146.1
**Gender assigned at birth**	Male	Male	Female	Male	Female	Male	Undecided	Undecided	Undecided
**Sex-of-rearing**	Male	Male	Male	Male	Male	Male	Male	Male	Male
**Repeated hospital admission for vomiting and/or seizures**	No	No	No	Yes	Yes	Yes	No	No	No
**Systolic blood pressure (mmHg)** **(percentiles)**	112 (p78)	135	157	112 (p92)	126 (p99)	122 (p94)	90 (p58)	100 (p81)	132 (p99)
**Diastolic blood pressure (mmHg) (percentiles)**	67 (p61)	94	108	72 (p88)	83 (p97)	90 (p99)	60 (p90)	70 (p95)	80 (p96)
Bone age (years)***	–	–	–	18.0	15.0	–	4.5	7.0	17.0
**Calendar age (years)**	–	–	–	10.1	7.9	–	3.5	4.5	11.9
**Bone age advancement (years)**	–	–	–	+7.9	+7.1	–	+1.0	+2.5	+5.1
**Tanner Stage**	M1P5	M1P5	M1P5	M1P5	M1P5	M1P5	M1P1	M1P1	M2P5
**Prader stage**	3	4	3	3	4	3	5	5	5
**Phallus length (cm)**	6.5	5.0	4.7	5.4	6.0	7.0	2.3	2.8	4.7
**Uterus present**	NK	NK	Yes	Yes	NK	NK	NK	NK	Yes
**Hyperpigmentation**	Yes	Yes	Yes	Yes	Yes	Yes	No	No	Yes
**Deepening voice**	Yes	Yes	Yes	Yes	Yes	Yes	No	Yes	Yes
**Acne**	No	Yes	Yes	Yes	Yes	Yes	No	No	Yes

*pair of siblings from non-consanguineous parents.

**three siblings from non-consanguineous parents.

***Bone age is measured at depicted calendar age.

Calculation of blood pressure percentiles for children from Flynn et al. (21).

NK, not known.

Four patients had a male sex assignment after birth, two were initially assigned female, and the remaining three had an undecided sex after birth. Of the two patients initially assigned as females at birth, one patient (patient 3) officially changed his gender at the age of 45, after experiencing gender dysphoria for a substantial period. The gender of the other patient (patient 5) was reassigned at age 3. This was a gradual transition initiated by the parents, partially guided by social pressure from the environment because of the male-like appearance of this child. At age 14, he is still satisfied with his registered male gender and lives as a boy. All included patients had a male sex-of-rearing at time of investigation.

Pubic hair was absent in two patients (3 and 5 years old), but the pubic hair of the other seven patients (aged 10 – 46 years old) was spread to the medial surface of the thighs (Tanner stage P5). Breast development was absent in all but one patient; one 11OHD patient (aged 13) had small breast buds with some stimulation of glandular tissue (Tanner stage M2). All patients showed virilization of the external genitalia with Prader stages ranging from 3 to 5. In three patients an ultrasound was performed which confirmed the presence of a uterus. Their phallus length varied from 2.3 to 7.0 cm. Hyperpigmentation, a sign of ACTH overproduction, was present in seven out of nine patients (78%), except in the two youngest patients (7 and 8). Deepening of the voice and acne were observed in eight (89%) and six (67%) patients, respectively. Three 21OHD patients reported one or recurrent episodes of hospitalization due to vomiting and/or seizures during infancy and childhood: patient 4 had only one reported episode at the age of 7 days old, patient 5 had eight reported episodes of seizures during childhood (at that time classified as febrile seizures), including one requiring intensive care stay, and patient 6 had seven periods of hospitalizations until the age of 5 years old, but not thereafter. Some hospitalization episodes were in local hospitals and the exact treatment regimen during hospital stay was unknown. None of the patients received daily glucocorticoid treatment on prescription after discharge from the hospital.

### 3.2 Genetical analysis

Genetic analysis confirmed the diagnosis of classic 21OHD (SW/SV) or 11OHD in all patients. The identified pathogenic variants and their predicted phenotype based on residual enzymatic activity in literature are shown in **Supplementary Table 1**. Some pathogenic variants were previously described (22). All patients had pathogenic variants with a predicted residual activity of <1%, except for patient 3, who had a pathogenic variant on one allele with 1 – 5% predicted residual activity. Based on genetic analysis, patients 1, 2 and 3 have a predicted SW phenotype, but reported no episodes of salt wasting. Therefore, these patients are phenotypically classified as SV.

### 3.3 Biochemical analysis

#### 3.3.1 Cortisol and precursor steroids

In **Table 2** the serum concentration of adrenal steroids before and 60 minutes after intravenous administration of ACTH are shown. The median unstimulated cortisol concentration in 21OHD and 11OHD patients was very low (89 nmol/L, IQR: 59 – 169) without increase after ACTH administration (median 0%, IQR -3.6% – 2.5%). In patients with 21OHD, unstimulated 17OHP concentrations were elevated (median 496 nmol/L, IQR: 445 – 820) compared to reference ranges with a further increase of 18.4% (IQR: -7% – 70%) after ACTH. The median concentration of 21-deoxycortisol (21DF) was 92.8 nmol/L (IQR: 46.6 – 126), which is highly elevated compared to the reference interval. For 11OHD patients, concentrations of 11-deoxycortisol and 11-deoxycorticosterone were elevated and increased further after ACTH stimulation (**Table 2**).

**Table 2:** Biochemical measurements (nmol/L) of patients before (basal) and after (post ACTH) an ACTH stimulation test.

Pt	Age	Gene	Phenotype	Cortisol		17OHP		11DF		P		21DF		B		11DB	
				Basal	Post ACTH	Basal	Post ACTH	Basal	Post ACTH	Basal	Post ACTH	Basal	Post ACTH	Basal	Post ACTH	Basal	Post ACTH
1	15	*CYP21A2*	SV	89	79	368	1100	1.03	1.12	6.82	42.8	73.6	200	4.78	12.20	0.12	0.27
2	32	*CYP21A2*	SV	61	65	991	1160	1.39	1.55	54.6	65.9	117	112	9.50	10.00	0.42	0.43
3	46	*CYP21A2*	SV	159	159	763	665	2.56	2.39	50.2	39.3	112	101	13.20	12.00	0.44	0.39
4	10	*CYP21A2*	SW	61	60	470	598	1.8	1.83	12.5	25.4	56.2	88.2	3.79	5.80	0.27	0.40
5	14	*CYP21A2*	SW	53	50	486	582	1.16	1.25	13.5	17.7	154	139	6.12	6.08	0.49	0.58
6	15	*CYP21A2*	SW	57	58	506	480	1.7	1.68	20.5	21.5	17.8	13.3	5.06	4.36	0.57	0.57
7	3	*CYP11B1*	Classic	202	202	13	22	417	567	2.17	5.58	<1.0	<1.0	5.76	8.80	55.5	124
8	5	*CYP11B1*	Classic	150	155	11	19	301	457	2.60	6.03	< 1.0	< 1.0	2.55	8.08	35.4	113
9	13	*CYP11B1*	Classic	179	180	6	20	196	657	0.74	3.57	< 1.0	< 1.0	1.03	3.56	9.97	55.3
Reference intervals*				**Cortisol**		**17OHP**		**11DF**		**P**		**21DF**		**B**		**11DB**	
Female**				190 – 550		0.45 – 3.8		0.2 – 4.3		<0.62		<0.7		1.8 – 34.4		<0.49	
**Male**				190 – 550		2.0 – 10.8		0.2 – 4.3		<0.47		<0.7		1.3 – 36.4		<0.49	
**Child**				190 – 550		0.2 – 7.4		0.2 – 4.3				<0.7				<0.49	

*Reference intervals are for basal conditions.

**Reference intervals for pre-menopausal women in follicular phase.

Pt, patient number; 17OHP, 17-hydroxyprogesterone; 11DF, 11-deoxycortisol; P, progesterone; 21DF, 21-deoxycortisol; B, corticosterone; 11DB, 11-deoxycorticosterone.

#### 3.3.2 Mineralocorticoids

Renin concentrations were elevated in all untreated 21OHD patients (range: 57 – 280 mU/L) compared to the reference interval (**Table 3**). The three patients with 11OHD had decreased or low-normal renin concentrations (<3, 5.9, and 7.9 mU/L). One of these patients (aged 13) had an elevated systolic blood pressure (132 mmHg) compared to the reference range for age. The two youngest 11OHD patients (3 and 5 years old) had a normal systolic blood pressure. All three 11OHD patients had an elevated diastolic blood pressure (≥p90) (**Table 1**).

**Table 3:** Biochemical description of renin levels and the gonadal axis in nine untreated 46,XX congenital adrenal hyperplasia patients with a male sex of rearing.

Pt	Age	Gene	Phenotype	Renin (mU/L)	LH (IU/L)	FSH (IU/L)	Estradiol (pmol/L)	Estrone (pmol/L)
**1**	15	*CYP21A2*	SV	140	3.81	4.66	101	569
**2**	32	*CYP21A2*	SV	61	5.77	5.17	216	1006
**3**	46	*CYP21A2*	SV	57	<0.3	<0.3	70.1	568
**4**	10	*CYP21A2*	SW	280	<0.3	<0.3	137	940
**5**	14	*CYP21A2*	SW	84	<0.3	<0.3	135	852
**6**	15	*CYP21A2*	SW	100	<0.3	<0.3	239	1525
**7**	3	*CYP11B1*	Classic	5.9	–	–	9.9	121
**8**	5	*CYP11B1*	Classic	<3	<0.3	1.1	11.5	165
**9**	13	*CYP11B1*	Classic	7.9	3.43	5.62	269	260
Reference intervals				Renin (mU/L)	LH (IU/L)	FSH (IU/L)	Estradiol (pmol/L)	Estrone (pmol/L)
Female*				6.2 – 51	2.4 – 12.6	3.5 – 12.5	31 – 771	15 – 492
**Male**				6.2 – 51	1.7 – 8.6	1.5 – 12.4	12 – 136	33 – 133
**Child**				6.2 – 51	<0.15 – 1.3	<0.15 – 3.7	<20	<96 (for girls)

*Reference intervals for pre-menopausal women (in early follicular phase if applicable).

Pt, patient number; LH, luteinizing hormone; FSH, follicle stimulating hormone.

#### 3.3.3 Pituitary-gonadal axis

The median estradiol and estrone concentrations of the adolescent and adult patients were 137 pmol/L (IQR 101 – 240) and 852 pmol/L (IQR 568 – 1006), respectively. Patient 7 and 8 (aged 3 and 5 years old) had estradiol concentrations <20 pmol/L, which are consistent with their prepubertal state. These patients also had the lowest estrone concentrations, of 121 and 165 pmol/L respectively, which is, despite the age of these patients, within the adult female reference range. The patient (aged 13) who presented with some breast development (Tanner stage M2) had the highest estradiol concentration (269 pmol/L). Concentrations of LH and FSH were <0.3 IU/L in four out of seven (57%) adolescent and adult patients indicating an inactive hypogonadotropic state. The three other patients (43%) had gonadotropin concentrations within the adult reference interval (**Table 3**).

#### 3.3.4 Androgens

**Figure 4:**
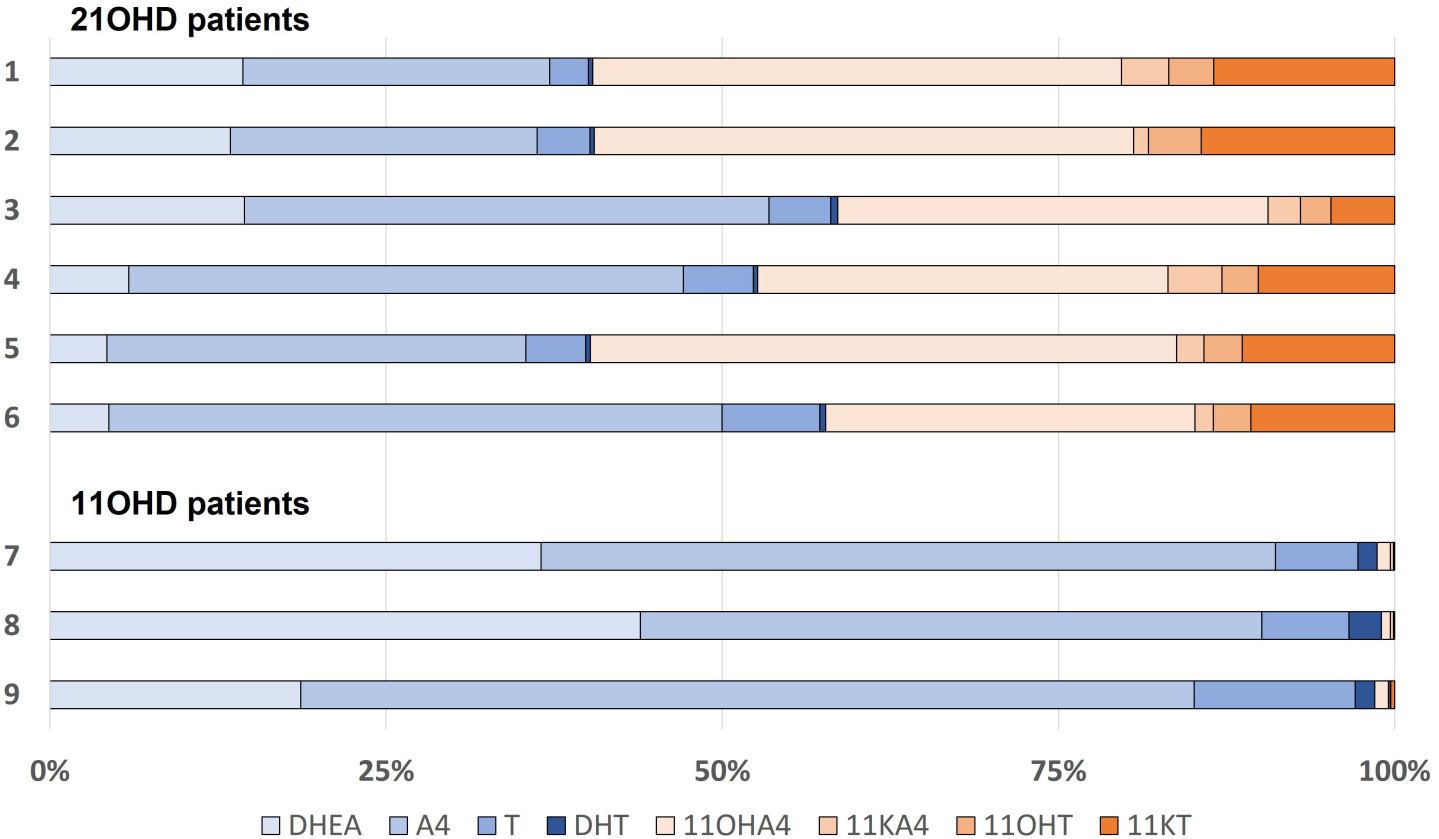
Relative androgen concentration in patients with 21-hydroxylase deficiency (21OHD; patient 1-6) or 11-hydroxylase deficiency (11OHD; patient 7-9). DHEA, dehydroepiandrosterone; A4, androstenedione; T, testosterone; DHT, dihydrotestosterone; 11OHA4, 11-hydroxyandrostenedione; 11KA4, 11-ketoandrostenedione; 11OHT, 11-hydroxytestosterone; 11KT, 11-ketotestosterone.

The concentration of DHEA were for al described patients within or just above the corresponding reference range. The median basal A4 concentration was 85 nmol/L (IQR 27.8 – 164) with a median increase of 5.5% (IQR -0.3 – 34.9) after ACTH stimulation. For T, the median basal concentration was 12.8 nmol/L (IQR 4.76 – 19.5) with a median increase of 6.5% (IQR -7.5 – 15.4). Serum testosterone is mostly bound to proteins such as SHBG. The median SHBG concentration was 23.6 nmol/L (IQR 21.1 – 58.4) (**Table 4**). The median free (i.e., unbound) T was 231.5 pmol/l (IQR 102 – 472), which is 2.1% (IQR 1.2 – 2.2) of the total T concentration. In the 21OHD patients (age range 10 – 46 years), median concentrations for 11OHA4, 11KA4, 11OHT, and 11KT were 127, 6.6, 10.7, and 41.8 nmol/L respectively and the 11-oxygenated androgens comprised 41 – 60% of the total measured androgen pool in these patients (**Figure 4**). Therewith, 11-oxygenated androgens contribute significantly to the total androgen pool. For all 21OHD patients, the 11KT concentration was higher than the T concentration. The ratio between A4, T, and their respective 11-oxygenated metabolites showed high variability among the 21OHD patients: 0.60 – 1.76 for 11OHA4/A4, 0.03 – 0.15 for 11KA4/A4, 0.38 – 1.17 for 11OHT/T, and 1.03 – 4.68 for 11KT/T (**Table 5**). For the 11OHD patients (aged 3, 6 and 13), concentrations of 11KA4, 11OHT, and 11KT were below or around the lower limit of quantification (respectively 0.09, 0.04, and 0.05 nmol/L). The 11OHA4 concentrations were 0.3, 0.3, and 0.5 nmol/L for patients 7, 8, and 9, respectively. In 11OHD patients, the 11-oxygenated androgens are 1.0 – 1.5% of the total measured androgen pool (**Figure 4**).

**Table 4:** Androgen concentrations and SHBG levels (nmol/L) of nine untreated 46,XX congenital adrenal hyperplasia patients with a male sex of rearing.

Pt	Age	Gene	Phenotype	DHEA	A4	T	Free T (%)	DHT	11OHA4	11KA4	11OHT	11KT	SHBG
1	15	*CYP21A2*	SV	43.9	69.8	8.8	2.0	0.9	120	10.7	10.3	41.2	22.9
2	32	*CYP21A2*	SV	48.7	82.8	14.3	2.1	1.1	146	3.9	14.3	52.2	20.8
3	46	*CYP21A2*	SV	40.2	108	12.8	1.1	1.5	88.8	6.7	6.2	13.2	62.2
4	10	*CYP21A2*	SW	25.9	182	22.9	2.2	1.4	135	17.7	11.8	44.7	24.2
5	14	*CYP21A2*	SW	13.5	99.4	14.2	2.2	1.2	139	6.5	9.1	36.1	18.1
6	15	*CYP21A2*	SW	17.5	181	28.9	2.3	1.7	109	5.4	11.1	42.5	21.9
7	3	*CYP11B1*	Classic	12.5	18.6	2.1	–	0.5	0.3	<0.09	<0.04	<0.05	–
8	5	*CYP11B1*	Classic	19.6	20.6	2.9	0.4	1.1	0.3	0.1	<0.04	<0.05	190
9	13	*CYP11B1*	Classic	9.9	35.2	6.4	1.3	0.8	0.5	<0.09	<0.04	0.143	47.1
Reference intervals				DHEA	A4	T		DHT	11OHA4	11KA4	11OHT	11KT	SHBG
Female*				2.1 – 41	0.9 – 7.5	0.5 – 2.0		0.1 – 0.86	3.4 – 14.0	1.6 – 5.4	<0.33 – 0.78	<0.33 – 1.6	32 - 128
**Male**				1.8 – 37	1.2 – 4.7	11 - 37		0.9 – 3.2	2.9 – 14.0	1.42 – 6.2	<0.33 – 1.1	<0.33 – 2.2	18 - 54
**Prepubertal girls**				<0.35 – 14	0.07 – 1.0	0.02 – 0.3		<0.1 - <0.1	0.21 – 3.7	<0.09 – 0.49	<0.04 – 0.15	<0.05 – 0.99	
**Prepubertal boys**				<0.35 – 14	0.03 – 1.1	0.02 – 0.3		<0.1 - <0.1	0.21 – 3.7	<0.09 – 0.49	<0.04 – 0.15	<0.05 – 0.99	

*Reference intervals for pre-menopausal women.

Reference intervals for 11-oxygenated androgens in children from Adriaansen et al. (5) and 5^th^ and 95^th^ percentiles in adults <50 years from Schiffer et al. (23).

Pt, patient number; DHEA, dehydroepiandrosterone; A4, androstenedione; T, testosterone; DHT, dihydrotestosterone; 11OHA4, 11-hydroxyandrostenedione; 11KA4, 11-ketoandrostenedione; 11OHT, 11-hydroxytestosterone; 11-KT, 11-ketotestosterone.

Concentrations below the lower limit of quantification (LLOQ) are denoted as ‘<LLOQ’.

**Table 5:** Androgen ratios of nine untreated 46,XX congenital adrenal hyperplasia patients with a male sex of rearing.

Pt	Age	Gene	Phenotype	A4/T	11OHA4/A4	11KA4/A4	11OHT/T	11KT/T
1	15	*CYP21A2*	SV	7.9	1.72	0.15	1.17	4.68
2	32	*CYP21A2*	SV	5.8	1.76	0.05	1.00	3.65
3	46	*CYP21A2*	SV	8.4	0.82	0.06	0.49	1.03
4	10	*CYP21A2*	SW	7.9	0.74	0.10	0.52	1.95
5	14	*CYP21A2*	SW	7.0	1.40	0.07	0.64	2.54
6	15	*CYP21A2*	SW	6.3	0.60	0.03	0.38	1.47
7	3	*CYP11B1*	Classic	8.9	–	–	–	–
8	5	*CYP11B1*	Classic	7.1	–	–	–	–
9	13	*CYP11B1*	Classic	5.5	–	–	–	–

Pt, patient number; A4, androstenedione; T, testosterone; 11OHA4, 11-hydroxyandrostenedione; 11KA4, 11-ketoandrostenedione; 11OHT, 11-hydroxytestosterone; 11-KT, 11-ketotestosterone.

## 4 Discussion

In this study, we provide a clinical and biochemical description of nine untreated 46,XX classic CAH patients with male sex-of-rearing. This is the first study investigating the steroid profile including 11-oxygenated androgens in untreated CAH patients. Here, we will discuss the cortisol and precursor steroids, mineralocorticoids, pituitary-gonadal axis, and androgens separately. Thereafter, we will discuss the challenges in treatment in male-reared 46,XX CAH patients.

### 4.1 Cortisol and precursor steroids

As expected, all 21OHD patients had strongly elevated 17OHP concentrations. The observed increased 21DF concentrations in the 21OHD patients result from conversion of overproduced 17OHP by *CYP11B1*. In some 21OHD patients, 17OHP concentrations did not rise after ACTH administration, suggesting that they already exhibited maximal enzymatic response to ACTH stimulation. This is confirmed by other studies in untreated CAH patients (24–26).

In our cohort of untreated CAH patients, the cortisol concentrations were low and did not significantly increase after ACTH administration as is expected in classic CAH patients. This indicates a potentially decreased capacity to adapt to physiological stress (27). Three 21OHD patients experienced periods of seizures and/or vomiting until the age of 6 years old, but not thereafter. This might be a sign of glucocorticoid and/or mineralocorticoid deficiency. Interestingly, patients were not diagnosed with CAH at that moment and they survived these periods without adequate adrenal crisis treatment. The other three 21OHD and classic 11OHD patients did not experience clinical symptoms of cortisol deficiency nor reported hospitalization periods, despite their low cortisol levels. The elevated levels of precursor steroids with glucocorticoid activity such as 21DF and 11-deoxycortisol might safeguard the glucocorticoid activity in these patients, which could explain the lack of signs of cortisol deficiency. Engels et al. (16) showed that 21DF and 11-deoxycortisol can transactivate the glucocorticoid receptor with a relative potency compared to cortisol of 49% and 15%, respectively. Furthermore, it can be speculated that measuring the total cortisol concentration does not optimally represent the biological glucocorticoid activity. According to the free hormone hypothesis, the free cortisol (i.e., unbound to proteins) rather than the total cortisol concentration affects the biological activity of cortisol. Another hypothesis may be the presence of crosstalk between the androgen and glucocorticoid pathways. Kroon et al. (28) described in their recent review that androgens crosstalk with the glucocorticoid pathway on various levels. Firstly, as androgen receptors and glucocorticoid receptors show high structural similarity, they may bind to the same responsive elements on the DNA. Secondly, androgens mediate the (re-)opening of the chromatic structure in DNA and hence facilitate glucocorticoid receptor signaling. Lastly, androgens regulate the expression of 11β-hydroxysteroid dehydrogenase type 1 in peripheral tissues. This enzyme locally catalyzes the conversion from inactive cortisone into active cortisol. CAH patients have elevated concentrations of androgens that might interact with the glucocorticoid pathway, enhancing its actions. This could be an additional explanation for the low occurrence of symptoms of cortisol deficiency in this cohort despite the low cortisol concentrations. However, it is important to note that we described a potentially biased subset of patients. Their glucocorticoid concentrations were sufficient to survive until now, but this may not be generalizable to all CAH patients as other unknown protective factors may play a role. There were no patients with a homozygous deletion in our cohort and, therefore, all included patients had some residual enzymatic activity. It is important to acknowledge that classic CAH patients are at risk of an adrenal crisis and withholding these patients glucocorticoid treatment may be lethal.

### 4.2 Mineralocorticoids

In our cohort, three untreated 21OHD patients with a salt-wasting phenotype reported recurrent episodes of vomiting and/or seizures during infancy. After the age of 6, they had no hospitalization episodes despite not receiving mineralocorticoid medication, which might be due to the high sodium intake from Indonesian food preventing them from salt wasting crisis (29).

Patients with 11OHD exhibit high serum levels of 11-deoxycorticosterone, which is known to have mineralocorticoid activity (30), leading to suppressed renin levels and hypertension as observed in the oldest 11OHD patient (aged 13). The two other 11OHD patients (aged 3 and 5) did not develop hypertension until now. Further follow-up is needed, as these patients are prone to develop hypertension at later age.

### 4.3 Pituitary-gonadal axis

Adolescent and adult 46,XX CAH patients with suppressed LH and FSH concentrations (<0.3 IU/L) had estradiol concentrations within the adult female reference range, indicating that these estrogens are produced via aromatization of testosterone rather than directly through ovarian biosynthesis. Estrone concentrations of all 21OHD patients were above the upper reference limit, which is most likely due to aromatization of increased levels of A4. In addition, these patients have increased adrenal progesterone production. Both estrogens and progesterone give negative feedback on the hypothalamus-pituitary-gonadal axis (31), explaining the observed hypogonadotropic state which prevents female pubertal development and menstruation. However, three patients had gonadotrophins within the adult reference range, suggesting less negative feedback from estrogens and progestogens to the pituitary gland. This might be due to interpersonal differences in receptor sensitivity. One 11OHD patient (aged 13) with the highest estradiol levels had some breast development. In this patient, the androgen concentrations were probably not high enough to suppress ovarian estrogen biosynthesis. The presence of a uterus was confirmed in three out of nine patients. In the remaining patients, the presence of a uterus and ovaries is assumed because all had a 46,XX genotype. However, none of the patients had a menstrual cycle, because the high androgen and estrogen concentrations have negative feedback on the hypothalamus-pituitary-gonadal axis, resulting in unstimulated ovaries and endometrium. It should be noted that accurately visualizing an unstimulated uterus or ovary by ultrasonography is challenging.

### 4.4 Adrenal androgens

In our study, the A4 and T concentrations in all adolescent and adult patients were within the normal range for healthy adult males, and higher compared to healthy female reference ranges. All patients with 21OHD also had increased concentrations of 11-oxygenated androgens. Concentrations of 11-oxygenated androgens were higher compared to the concentrations of A4 and T. The agonistic activity on the androgen receptor of 11KT is similar to T (6–8). So, the 11-oxygenated androgens contribute significantly to the total androgen pool both in quantity and the potency to activate the androgen receptor. Therefore, merely measuring A4 or T in routine clinical management does not reflect the total androgen activity in CAH patients. Additional quantification of 11-oxygenated androgens in 21OHD patients will give a more comprehensive insight into serum androgen status in these patients. Some patients with 11OHD were still able to produce minute quantities of 11-oxygenated androgens, suggesting the presence of residual enzymatic activity. For these patients, it would be interesting if intermediates of the backdoor pathway could be measured in future research to further elucidate the origin of the elevated androgen concentrations.

### 4.5 Challenges in treatment

International guidelines used to recommend early genital surgery in virilized girls with CAH (32), but nowadays genital surgery is often performed later in life and in some countries prohibited at young age (1). For sex assignment in neonates with differences of sex development, shared-decision making and transparent informed consent procedures are very important in the counseling process (33). It has been recently published that male-reared 46,XX CAH patients can successfully establish a male gender identity or role with an overall good quality of life (34). The patients in our study also successfully established a male gender role, but conformity bias cannot be excluded. There is a challenge in treating 46,XX CAH patients with a male sex of rearing; These patients have strongly elevated adrenal androgen concentrations and therefore, their external genitals may appear male. Preserving these high adrenal androgen concentrations is desired to maintain their male appearance and prevent the formation of female puberty with breast development and menstruation. However, untreated classic CAH patients are potentially susceptible to developing symptoms of cortisol deficiency and are at risk for developing an Addisonian crisis. Therefore, it is recommended to treat classic CAH patients with glucocorticoids (11). This substitution therapy will also lead to a decline in ACTH-driven adrenal androgen production. Moreover, this leads to a decrease of progesterone levels during puberty, and the hypothalamus-pituitary-gonadal axis will be activated, initiating ovarian production of estrogens and consequently female breast development. In these 46,XX male-reared patients, decreasing the androgen concentrations is undesired. All patients were counseled about glucocorticoid medication but decided not to start this medication due to these effects and the lack of complaints in daily life. Previously, two case series described 46,XX CAH patients with male gender identity who received testosterone treatment in addition to their glucocorticoid treatment (13, 14). This allows correction of low cortisol levels while maintaining androgen concentrations within the male range. In Indonesia, testosterone treatment is mainly prescribed in 46,XY male patients with hypogonadism and is generally not used for other indications. To prevent adrenal crisis events, patients should be given sick day rule advice with glucocorticoid stress dosing during periods of physical stress.

To conclude, we described a unique group of nine untreated 46,XX male classic CAH patients who survived without glucocorticoid treatment despite low cortisol concentrations. All patients had androgen levels within the male reference range and elevated 11-oxygenated androgens that contribute significantly to the total androgen pool in 21OHD patients. Therefore, 11-oxygenated androgens should be considered during routine clinical management in 21OHD patients to give a more comprehensive insight into their adrenal androgen status. Treatment of these patients is challenging as the most common treatment with glucocorticoids will lead to a decrease in their ACTH-driven androgen production. This will eventually lead to the development of female puberty including menstruation, which is undesirable for these patients. We note that this is a potentially biased group, and we cannot exclude that other classic CAH patients passed away before diagnosis. Therefore, we do not advise withholding glucocorticoid treatment in classic CAH patients as they are always at risk for an adrenal crisis. An individual treatment approach with careful counseling about their hormonal treatment is needed for these patients. If patients do not prefer glucocorticoid treatment, they should be counseled about the risks.

## Data availability statement

The original contributions presented in the study are included in the article/**Supplementary material**. Further inquiries can be directed to the corresponding author.

## Ethics statement

The studies involving humans were approved by Ethical committee of the faculty of medicine, Diponegoro University, Semarang, Indonesia (number 713/EC/FK-RSDK/2016). The studies were conducted in accordance with the local legislation and institutional requirements. Written informed consent for participation in this study was provided by the participants’ legal guardians/next of kin. Written informed consent was obtained from the individual(s), and minor(s)’ legal guardian/next of kin, for the publication of any potentially identifiable images or data included in this article.

## Author contributions

BA: Conceptualization, Data curation, Formal analysis, Investigation, Methodology, Project administration, Resources, Software, Validation, Visualization, Writing – original draft, Writing – review & editing. AU: Conceptualization, Data curation, Funding acquisition, Investigation, Methodology, Project administration, Software, Writing – original draft, Writing – review & editing. DW: Formal analysis, Investigation, Methodology, Resources, Writing – original draft, Writing – review & editing. AJ: Writing – original draft, Writing – review & editing. MA: Writing – original draft, Writing – review & editing. AE: Writing – original draft, Writing – review & editing. MS: Writing – original draft, Writing – review & editing. PS: Writing – original draft, Writing – review & editing, Supervision, Resources. FS: Writing – original draft, Writing – review & editing, Supervision, Resources. SD: Writing – original draft, Writing – review & editing, Supervision, Resources. SF: Writing – original draft, Writing – review & editing, Supervision. AvH: Writing – original draft, Writing – review & editing, Supervision, Resources. HC-vdG: Writing – original draft, Writing – review & editing, Supervision, Conceptualization, Resources.
