# Supplementary material for: 46,XX males with congenital adrenal hyperplasia: a clinical and biochemical description
Source: Front Endocrinol (Lausanne). 2024 Aug 8;15:1410122. doi: 10.3389/fendo.2024.1410122 (PMC11338787; doi:10.3389/fendo.2024.1410122)
Supplement: Supplementary file 1 [file Table_1.docx]

Supplementary Material

***Supplementary Table 1*** *Identified pathogenic variants in the CYP21A2 gene (NM_000500.9) and the CYP11B1 gene (NM_000497.3). For the respective variant combinations, the predicted phenotype based on residual enzyme activity and literature (for CYP21A2: [19]). Adherence to HGVS nomenclature rules (varnomen.hgvs.org) makes the nucleotide numbers 3 higher and the amino acid numbers 1 higher than the legacy names used in literature (for CYP21A2).*

| **Pt** | **Gene** | **Variant 1** | | | |  | **Variant 2** | | | | **Assumed inheritance** | **Predicted phenotype**  **based on genotype** | **Clinical phenotype** |
| --- | --- | --- | --- | --- | --- | --- | --- | --- | --- | --- | --- | --- | --- |
|  |  | **Nucleotide change** | **Protein change** | **Legacy name** | **(Predicted) enzyme activity** |  | **Nucleotide change** | **Protein change** | **Legacy name** | **(Predicted) enzyme activity** |  |  |  |
| 1 | *CYP21A2* | c.293-13A/C>G | r.spl? p.? | I2G | 0-1% |  | c.293-13A/C>G | r.spl? p.? | I2G | 0-1% | homozygous | SW | SV |
| 2 | *CYP21A2* | c.293-13A/C>G | r.spl? p.? | I2G | 0-1% |  | c.293-13A/C>G | r.spl? p.? | I2G | 0-1% | homozygous | SW | SV |
| 3 | *CYP21A2* | c.60G>A | p.(Trp20*) | W19X | 0% |  | c.518T>A | p.(Ile173Asn) | I172N | 1-5% | compound heterozygous | SW/SV | SV |
| 4 | *CYP21A2* | c.586C>T | p.(Gln196*) | unknown | 0% |  | c.1069C>T | p.(Arg357Trp) | R356W | 0% | compound heterozygous | SW | SW |
| 5 | *CYP21A2* | c.1155_1157del | p.(Ile386del) | unknown | unknown |  | c.1069C>T | p.(Arg357Trp) | R356W | 0% | compound heterozygous | unknown | SW |
| 6 | *CYP21A2* | c.(?_-107)  (447+?)del | p.0 | deletion exon 1-3 | 0% |  | c.1069C>T | p.(Arg357Trp) | R356W | 0% | compound heterozygous | SW | SW |
| 7 | *CYP11B1* | c.799G>A | p.(Val252fs) | - | 0% |  | c.799G>A | p.(Val252fs) | - | 0% | homozygous | classic | classic |
| 8 | *CYP11B1* | c.799G>A | p.(Val252fs) | - | 0% |  | c.799G>A | p.(Val252fs) | - | 0% | homozygous | classic | classic |
| 9 | *CYP11B1* | c.799G>A | p.(Val252fs) | - | 0% |  | c.799G>A | p.(Val252fs) | - | 0% | homozygous | classic | classic |
